# Reexamining Feeding Tube Safety in Pediatrics: A Safety Event Rooted in Device Design and Instruction Gaps

**DOI:** 10.1097/pq9.0000000000000873

**Published:** 2026-02-24

**Authors:** Christopher J. Demas, RoseAnn Dubke, Rebecca A. Pehovic, Katherine E. Bates

**Affiliations:** From the * Department of Pediatrics, Corewell Health Helen DeVos Children’s Hospital, Grand Rapids, Mich.; † Department of Nursing, Mott Children’s Hospital of the University of Michigan, Ann Arbor, Michigan, and; ‡ Department of Cardiology, Mott Children’s Hospital of the University of Michigan, Ann Arbor, Michigan.

## Abstract

**Introduction:**

Nasogastric (NG) tubes are commonly used in hospitalized infants and children to provide nutrition and medications. Although clinical protocols emphasize the importance of confirming placement and tube patency, they pay less attention to the mechanical limits of NG tubes and the risks of fracture or rupture. Inconsistent guidance regarding tube care may contribute to preventable harm, particularly in the techniques used to clear obstructions.

**Methods:**

We presented a case of a newborn who experienced irreversible harm following an NG tube fracture or rupture. This event prompted a multidisciplinary review and simulated evaluation of NG tube performance using varying syringe sizes to mirror common bedside practices.

**Results:**

Simulation testing demonstrated that small-volume syringes, particularly 1–3 mL, can generate pressures high enough to balloon or rupture NG tubes. In contrast, larger syringes (≥30 mL) did not cause damage, even under maximum force. At the time of these events, manufacturer instructions did not provide pediatric-specific guidance regarding syringe selection, and local protocols additionally lacked specific recommendations. The size of the syringe selected may have contributed to tube fracture, esophageal rupture, and subsequent patient death.

**Conclusions:**

This case highlighted a safety gap in pediatric NG tube care, stemming from device performance and inconsistent instructional guidance. Improved alignment between manufacturer instructions and clinical resources, as well as pediatric-specific safety protocols, is essential to prevent similar events.

## INTRODUCTION

Temporary feeding tubes are essential for delivering enteral nutrition and medications to pediatric patients, particularly hospitalized infants and children or those unable to feed orally. These include nasogastric (NG), nasoduodenal, and nasojejunal tubes, which often use the same catheter, with the distinction based on the final position of the tube. In the United States, more than 1.2 million NG and nasoenteric tubes are placed annually.^1^ Pediatric data are limited, but children represent a substantial proportion of recipients: one 2014 study found 25% were children, including 6% younger than 12 months.^2^ A separate point-prevalence study reported that nearly 1 in 4 hospitalized children had temporary enteral access.^2^ In 2013, an estimated 190,000 children received enteral nutrition at home.^3^ Given their frequent use across both inpatient and outpatient settings, even uncommon complications can pose significant safety concerns for pediatric patients.

Complications related to NG tube use include both misplacement and device malfunction. Misplacement can lead to serious outcomes such as tracheobronchial feeding, pneumothorax, chemical pneumonitis, and acute respiratory distress.^4,5^ Device-related problems are also common; tube occlusion is a frequent issue, with reported clogging rates of 10%–23%.^6^ In many cases, improper flushing techniques, viscous feeding formulas, and medications that are inadequately prepared or cannot be administered in an ideal way contribute to tube occlusion.

Adding to these risks is a lack of pediatric-specific guidance in NG tube care. Many manufacturer instructions are tailored to adult patients and fail to account for pediatric anatomy or dosing considerations. As NG tube designs have evolved, newer models have removed features, such as backflow valves, which may inadvertently increase the risk of in situ tube fracture or rupture. These design changes, combined with adult-focused protocols, raise important questions about how to ensure safe and effective use of NG tubes in pediatric populations. This article described an event involving an NG tube rupture at our institution. It outlines a quality improvement initiative to identify contributing factors and implement system-level changes to prevent NG tube fractures or ruptures in pediatric patients.

## CASE PRESENTATION(S)

A newborn with 2 known genetic disorders was initially admitted with hyperammonemia and stabilized in the intensive care unit. Upon transfer to the general pediatric floor, the care team removed the patient’s 8-Fr NG tube due to a developing pressure injury and placed a 6-Fr NG tube without difficulty. The nurse confirmed placement by assessing gastric contents and verifying placement with pH measurement. During routine medication administration, the tube became clogged. Attempts to clear it with a 1-mL syringe flush of warm water, followed by a cola product flush, seemed successful.

Shortly after restarting feeds, the patient became pale and agitated. The nurse stopped the feeding and notified the provider. A chest x-ray revealed a discontinuity in the NG tube. When the nurse removed the NG tube, the distal portion remained in the patient and was subsequently successfully retrieved via urgent esophagogastroduodenoscopy. Soon afterward, the patient developed a pneumothorax, requiring chest tube placement and intensive care unit transfer. Over the next day, the patient’s respiratory distress worsened, necessitating intubation. The patient developed severe sepsis with Gram-negative bacteremia and, despite maximal support, passed away. Autopsy revealed esophageal rupture, likely secondary to an NG tube fracture.

## METHODS

At our hospital, this case was the third safety event submitted in 2 weeks regarding ballooning and rupturing of a 6-Fr NG tube. All 3 tubes were the same brand and size, suggesting a product defect. To mitigate the risk of continued malfunction, our hospital system promptly removed the involved NG tube (Avanos CORFLO 6 Fr) from stock and shared these concerns with the manufacturer.

Given the clustering of these events, a multidisciplinary group was assembled (attending physicians, fellows, residents, nurses, pharmacists, and patient safety specialists) to review all 3 cases to determine root causes. Local leaders provided support to staff involved in the incident, including second-victim support resources. During the interview process, the safety team discovered that all 3 tube malfunctions occurred shortly after unclogging the tubes with a 1-ml syringe. At the time, it was unclear if this was significant or merely coincidental.

To test whether syringe size contributed, our team conducted simulations across multiple NG tube sizes and brands (6- and 8-Fr feeding tubes from both Avanos CORFLO and NeoMed). A total of 14 tubes were warmed by submersion in body-temperature water, clamped to simulate occlusion, and flushed with syringes of different volumes by a single user.

## RESULTS

When attempting to unclog with a 35-mL syringe, the tubes remained intact despite the user applying maximal force. Sequential testing with progressively smaller syringes showed that using a 3-mL syringe generated sufficient pressure to cause tube ballooning and, in repeated trials, complete rupture. (See Video [online].)

**Video 1.** Feeding tube simulation testing

After simulation testing demonstrated that small-barrel syringes (1–3 mL) could cause ballooning or rupture across multiple NG tube sizes and brands, the team updated risk mitigation strategies. The initially removed product was returned to stock. With completion of the review, we updated the policy to require the use of 10 mL or larger syringes for routine flushing and mandate that declogging be performed only with 30 mL or larger syringes. Educational interventions, including simulation videos demonstrating NG tube rupture, have been implemented for both new-hire and existing adult and pediatric nursing staff to reinforce safe practices and standardize enteral tube care.

## DISCUSSION

NG tube rupture represents an underrecognized yet potentially devastating complication of enteral access, particularly in neonatal and pediatric patients. Similar risks are described in central venous catheter care, where smaller volume syringes produce higher pressures that have been linked to catheter damage or rupture.^7–9^ Although prior enteral nutrition guidelines caution against excessive pressure during declogging and note that feeding tube rupture has occurred, these statements are largely advisory and lack detailed descriptions of patient harm or contributing factors.^10,11^ To our knowledge, no published reports describe in situ NG tube rupture with associated patient harm or a subsequent systems-based analysis.

**Fig. 1.**
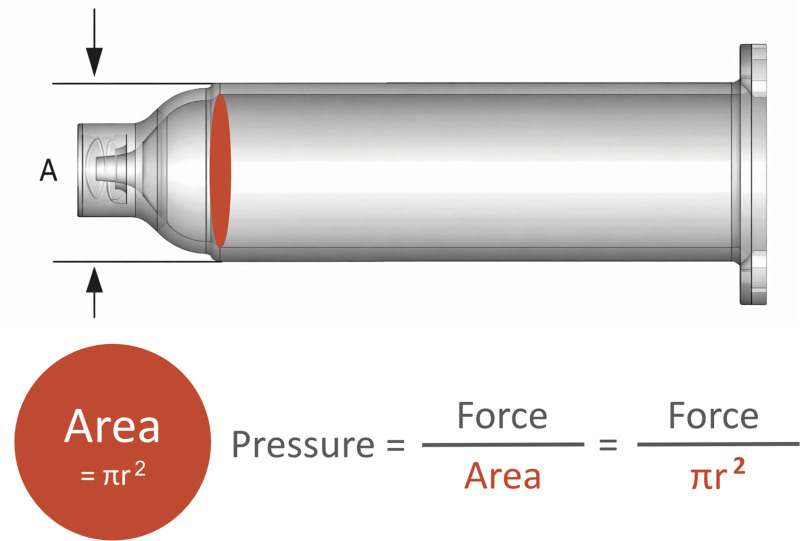
Schematic of a syringe used for NG tube flushing. Distance *A* represents the barrel diameter (twice the radius). The cross-sectional area of the barrel is a circle (π*r*^2^). Syringe pressure equals force/area, or force/π*r*^2^.

Fluid dynamics best explains the mechanism underlying this complication. Pressure generated during flushing is inversely related to the cross-sectional area of the syringe barrel: as syringe size decreases, the same applied force results in exponentially higher pressures (Fig. 1). This reasoning explains why small syringes (eg, 1–3 mL) can generate forces sufficient to balloon or rupture NG tubing, whereas larger syringes (≥30 mL) distribute force over a wider area and therefore generate substantially less pressure. For example, as shown in Table 1, applying the same amount of force with a 1-mL syringe produces more than 28 times the pressure generated with a 35-mL syringe. At the time of this event, the manufacturer did not provide pediatric-specific recommendations for syringe selection during declogging. Updated guidance published in December 2024 now specifies that clinicians should use only syringes of 30 mL or larger.^12^

**Table 1.** Relationship Among Syringe Size, Barrel Radius, and Relative Pressure Generated During Flushing

Syringe Size, mL	Radius, mm	Relative Pressure
35	24	1×
12	16	2.25×
6	13	3.41×
3	9	7.11×
1	4.5	28.44×

Because pressure is inversely related to the cross-sectional area (π*r*^2^), smaller syringe radii produce exponentially higher pressures.

Modern NG tube designs may further increase some risk. The new NG tube ENFit connectors were introduced to reduce the risk of intravenous and enteral medication misconnections; however, this design change also eliminated external pressure-release points. As a result, excessive pressure from an obstruction is directed into the patient. For neonates and small children whose tissues and anatomical structures are more fragile, this design vulnerability increases the risk of severe or even fatal outcomes when rupture occurs.

Adding to these design concerns, misconceptions about NG tube care continue to circulate in clinical practice, including the use of cola products or other beverages to clear occlusions. Although some of these practices are rooted in anecdotal experience, most lack pediatric-specific evidence and may introduce additional risks. Education must therefore address not only recommended practices but also unsafe practices to avoid, along with the rationale behind both.

This case also raised a broader institutional dilemma. Hospital systems rely on thousands of medical devices, each accompanied by manufacturer instructions or instructions for use (IFU). These documents may be updated over time, introduced with new product adoption, or lack pediatric-specific guidance. Although a comprehensive, ongoing review of every IFU presents clear operational challenges, misalignment between IFUs and institutional protocols can directly contribute to patient harm. This concern raises several important questions: What is an institution’s responsibility to ensure internal guidelines align with IFUs, and how should IFU review be prioritized across healthcare systems? A reasonable approach may be to focus first on devices that (1) are used frequently across broad patient populations, (2) have design features that increase risk in vulnerable groups (eg, pediatrics, neonates), or (3) are implicated in safety events or near misses. Equally important, device failures, especially when recurrent, should prompt bedside investigation and direct observation of real-world use to uncover practical causes of malfunction that may not be apparent from written protocols or IFUs. At the same time, protocol changes alone may not fully address the problem, as the device’s core design elements must also be considered. NG tubes may not intuitively seem high risk until events such as these reveal hidden vulnerabilities.

As a case report, this study provided limited insight into the long-term sustainability or generalizability of practice changes. Future work should extend these findings through a structured quality improvement approach with measurable aims, including monitoring NG tube-related safety events, compliance with syringe selection guidelines, and staff and caregiver education. Such efforts may support sustained practice change and facilitate dissemination through pediatric safety networks.

## CONCLUSIONS

This case underscores that even routine clinical practices (ie, unclogging a feeding tube) can result in irreversible harm when pediatric-specific factors are not fully considered. Although temporary feeding tubes remain essential tools in pediatric care, their safe use depends on a combination of appropriate device design, manufacturer guidance, institutional protocols, and frontline education.

More broadly, this case underscored the importance of sharing safety lessons broadly. Health systems should evaluate how internal guidelines align with manufacturer IFUs, particularly for devices used in vulnerable populations. Device failures, especially when recurrent, should prompt bedside observation and careful review to uncover practical risks that may not be evident from protocols alone.

By highlighting both device- and system-level factors, this report aimed to prompt other institutions to assess their own enteral tube practices, systematically review IFUs, and consider pediatric-specific adaptations. By sharing these lessons, we aim to prevent similar events and to foster broader collaboration among clinicians, safety teams, and device manufacturers to strengthen the systems that support safe, high-quality care for our most vulnerable patients.
